# X-ray lens figure errors retrieved by deep learning from several beam intensity images

**DOI:** 10.1107/S1600577524004958

**Published:** 2024-07-23

**Authors:** Manuel Sanchez del Rio, Rafael Celestre, Juan Reyes-Herrera

**Affiliations:** a European Synchrotron Radiation Facility, 71 Avenue des Martyrs, 38000Grenoble, France; Deutsches Elektronen-Synchrotron, Germany

**Keywords:** neural network, machine learning, compound refractive lens, modeling, simulation

## Abstract

The phase problem in the context of focusing synchrotron beams with X-ray lenses is addressed. The feasibility of retrieving the surface error of a lens system by using only the intensity of the propagated beam at several distances is demonstrated. A neural network, trained with a few thousand simulations using random errors, can predict accurately the lens error profile that accounts for all aberrations. It demonstrates the feasibility of routinely measuring the aberrations induced by an X-ray lens, or another optical system, using only a few intensity images.

## Abstract 2

A neural network trained with a few thousand simulations using random errors is demonstrated to predict accurately the lens error profile that accounts for all aberrations of a compound refractive lens in a synchrotron beamline.

## 1. Introduction

It is well known that the performances of the optical elements in a synchrotron beamline are limited by surface errors (Yabashi *et al.*, 2014 ▸; Schroer & Falkenberg, 2014 ▸; Cocco *et al.*, 2022 ▸). Surface errors originate aberrations in the X-ray beam, modifying its properties and limiting the required capabilities (usually focusing, but also affecting collimation and even energy resolution). Moreover, with the advent of a new generation of storage-ring sources (like the EBS-ESRF) and X-ray free-electron lasers, the requirements on the optics are more and more demanding, as beam quality (small emittance, beam coherence) dramatically improves. Now, beam degradation originating from any defect (either from fabrication, surface errors or from contamination) is amplified with a coherent source. The optics must accomplish its role in preserving the good qualities of the wavefront (Cocco *et al.*, 2022 ▸).

Whenever possible, surface errors must be minimized during the fabrication process. After fabrication, they are measured by metrology laboratories available at most synchrotron facilities – see, for example, Assoufid *et al.* (2005 ▸) and Rommeveaux *et al.* (2005 ▸, 2007 ▸). This *ex situ* characterization is fundamental for acceptance of the optics before installation in the beamline, but in many cases is not sufficient to determine the aberrations that will be produced in the X-ray beam. This is because the optical elements are dynamic and can move (*e.g.* bendable mirrors) or their configuration changes (*e.g.* transfocators that use a large number of X-ray lenses). Moreover, it is always useful to measure the errors using the same probe (light wavelength) that will be used in operation: X-rays. It is therefore convenient to make *in situ* (or at-wavelength) beamline measurements for the characterization of optical errors. This can be done using direct or indirect measurements. Examples of direct methods are wavefront sensors (Mercere *et al.*, 2005 ▸; Mikhaylov *et al.*, 2020 ▸) or X-ray phase-sensitive techniques such as speckle-tracking (Berujon *et al.*, 2020*a* ▸,*b* ▸) or ptychography (Schropp *et al.*, 2013 ▸). All of them require a dedicated infrastructure and experimental plan to retrieve the phase of the wavefront that encodes the information of the surface deformation profile (thus the errors). That means solving the ‘phase problem’, *i.e.* to determine the phase of a wave based on measurements of its intensity.

Indirect methods retrieve the wavefront aberration from the intensity images of a good quality beam, without using a specific instrument or technique. Examples of indirect measurements are the retrieval of an optical surface using images from a scanned slit (Zhang *et al.*, 2013 ▸), or looking at the beam evolution recording images at different positions like the beam caustics presented by Celestre *et al.* (2022 ▸). Indirect measurements of surface errors are much less accurate than direct *in situ* measurements or characterization from metrology laboratories but have the advantage that they can be obtained in a short time using only instrumentation available at the beamline. They are also limited in spatial resolution (highest spatial frequency), which depends on the quality of the beam used as a probe and the detector point spread function. However, the main problem with indirect measurements is the difficulty obtaining a good representation of the surface deformation profile (thus the errors) from intensity images. The optical surface errors are encoded in the wavefront phase, which is not directly measurable. The aberrations are related to wavefront deformation, therefore produced by surface errors and also encoded in the wavefront phase. Phase retrieval, or determination of the phase, requires the use of different techniques and algorithms, and several intensity images.

We analyze here an optical system to focus a synchrotron beam using X-ray lenses. It is derived from a typical beamline configuration at ESRF, in particular for ID18. This system was the object of previous investigations (Sanchez del Rio *et al.*, 2022 ▸). We solve the problem of phase retrieval from a collection of images measured at different distances around the focus by using a trained neural network. This work, based on simulations, demonstrates the feasibility of measuring the aberrations originated by the X-ray lenses using only intensity measurements in the neighborhood of the focal position. For that, we train a convolutional neural network (CNN) with synthetic profiles obtained from random variations of some generation polynomials. The shape of these profiles is compatible with what is generally found for embossed 2D lenses (Roth *et al.*, 2017 ▸) regardless of their radius or curvature (Celestre *et al.*, 2020 ▸, 2022 ▸; Seiboth *et al.*, 2020 ▸; Dhamgaye *et al.*, 2020 ▸).

Machine learning is ubiquitous and used in large facilities, like in tokamaks (Degrave *et al.*, 2022 ▸), accelerator control (Edelen *et al.*, 2020 ▸) for synchrotron radiation, setting insertion device parameters (Sheppard *et al.*, 2022 ▸), *etc*. It is also quite popular for the analysis of aberrations with wavefront sensors (Nishizaki *et al.*, 2019 ▸; Möckl *et al.*, 2019 ▸; Vanberg *et al.*, 2019 ▸). In particular, the analysis of aberrations with deep learning for 3D microscopy applications (Saha *et al.*, 2020 ▸) has inspired and guided us in our research.

## 2. Methods

### 2.1. Description of the optical system

The optical configuration studied here consists of a single X-ray lens illuminated by a monochromatic X-ray beam emitted by an undulator in the EBS-ESRF storage ring. It is a simplified part of the future EBSL1 beamline. The X-ray source is a U18 undulator (period λ_u_ = 18 mm) with *N*_u_ = 138 periods placed in the center of a straight section of the EBS storage ring.^1^ The gap is tuned to have the first harmonic at *E* = 7 keV (deflecting parameter *K* = 1.851). We consider a Be compound refractive lens (CRL) with parabolic profile and overlapping error profile (described later). The lens is located at a distance *p* = 65 m from the source. A first CRL implements a single lens with radius at the apex *R* = 50 µm (*f* = 3.952 m at 7 keV), and the aperture (diameter) is *a* = 1500 µm. A second CRL tested uses ten lenses of the same radius and aperture, therefore with a focal distance ten times shorter. Observation screens are placed in the vicinity of the geometrical focus, at an approximated distance *f* downstream of the lens.

The beamline also included other elements that, for simplicity, are not simulated. Two pairs of slits are not included. The first one at 36 m from the source (primary slits) selects the central cone of the undulator or part of it. We directly simulate the full central cone, therefore this slit is not needed. Another set of slits is at 65 m from the source (CRL entrance slit). It is considered fully open. The beamline CRL is for a monochromatic beam, meaning that a double-crystal monochromator (DCM) is placed upstream from the CRL. The typical Si 111 DCM has a resolution of approximately Δ*E*/*E* ≃ 10^−4^, therefore less than 1 eV at the used energy of 7 keV. The chromatic aberrations within this small bandwidth are negligible; therefore it is reasonable to use strictly monochromatic wavefronts, as we do. In theory, the monochromator does not modify the focusing if the crystals have the ideal (plane) optical surfaces. However, the thermal load makes the surfaces non-planar, thus introducing aberrations. The monochromators are designed to minimize these errors to limits to produce an irrelevant loss in energy resolution that is typically accompanied by no change in focusing. In the eventual case that there is some residual curvature, it would mostly affect the radius of curvature (defocus, which can be corrected) and not the other aberrations with higher spatial frequency.

### 2.2. One-dimensional wavefront model of the system

**Figure 1:**
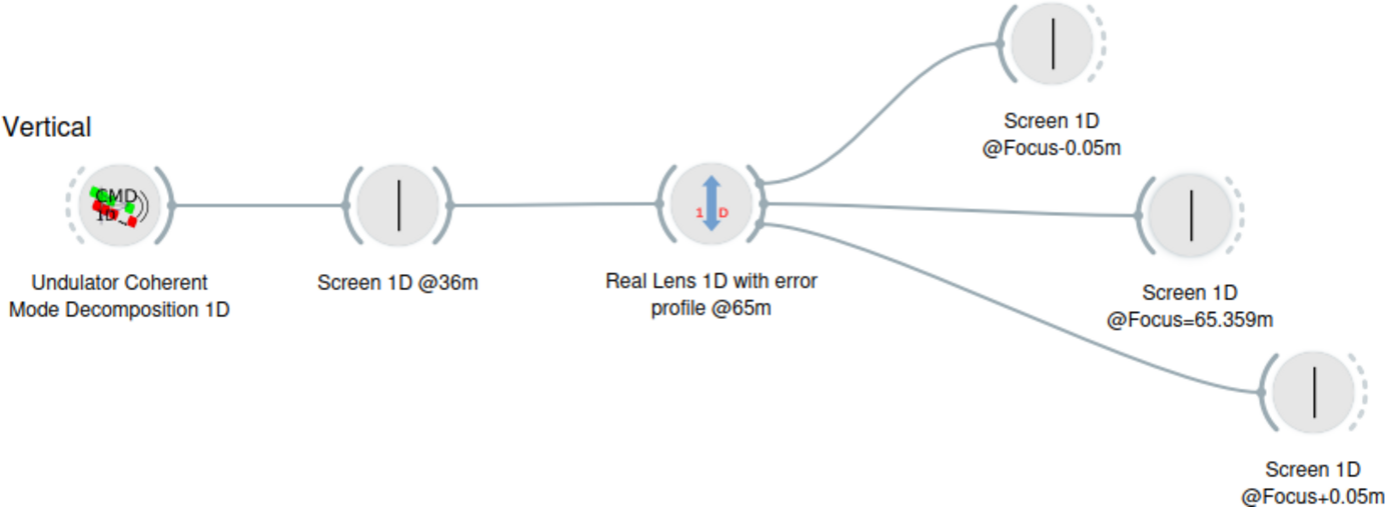
*OASYS* workspace containing a flowchart with a single beamline simulation.

The complexity of modeling accurately and realistically a synchrotron system resides in the fact that the beam is partially coherent. Completely incoherent beams can be simulated using ray tracing, and fully coherent beams with wavefront propagation. Partial coherence uses wavefront simulations, but considering multiple wavefronts. Two methods are used: multielectron Monte Carlo simulation (Chubar *et al.*, 2011 ▸) and coherent mode decomposition (Glass & Sanchez del Rio, 2017 ▸). We recently discussed an interest in studying the system in one dimension and demonstrated the possibility of performing coherent-mode decomposition (Sanchez del Rio *et al.*, 2022 ▸) using fewer computer resources. We decided to first use this 1D method to study the system in the vertical direction. The intensity distribution of the refracted beam is recorded at *N*_P_ different propagation positions (downstream from the lens) in an interval around the focal length *f* ± Δ*d*, which for the single-lens CRL is 3.592 m ± 0.5 m and for the multi-lens CRL is 0.359 m ± 0.05 m. The CRL is simulated by applying the thin element approximation (Celestre *et al.*, 2020 ▸; Sanchez del Rio *et al.*, 2022 ▸), using the cumulated profile of the CRL (adding one or ten parabolic profiles for the single-lens CRL and the multi-lens CRL, respectively) plus the error profile (that considers the cumulated error of all lens interfaces). The *OASYS* (Rebuffi & Sanchez del Rio, 2017 ▸) simulation workflow is shown in Fig. 1 ▸.

#### 2.2.1. Sampling error profiles

In a simulation, a thin layer of the lens material (Be) with a given profile is added to the parabolic profile of the lens. Lens refraction is simulated using the thin object approximation [see, for example, Celestre *et al.* (2020 ▸) and Sanchez del Rio *et al.* (2022 ▸)]. Our main objective is to retrieve this profile from the refracted beam intensities. In the thin element approximation, the error profile in projection approximation Δ_*z*_ is directly proportional to the phase ϕ it impinges on the wavefront: 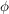 = 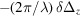, where λ is the wavelength and δ is the index of refraction decrement as in*n* = 1 − δ. Hence, obtaining the error profile from intensity measurements can be seen as a way of addressing the phase problem (Taylor, 1981 ▸; Klibanov *et al.*, 1995 ▸). To do that, we will train a CNN, but for that we need a large collection of lens error profiles. We describe here how to parametrize and sample the error profiles to have realistic sampled data. In terms of machine learning [see, for example, Chollet (2017 ▸)], this is part of the feature engineering, a process of using your knowledge about the data and the CNN to make the algorithm work better by applying hardcoded (non-learned) transformations to the data before it goes into the model. Our experience measuring and analyzing 2D lens profiles indicated that, although their topography looks complex, they can be fitted with great accuracy using Zernike polynomials – see comparisons in Fig. 5 to 8 of Celestre *et al.* (2020 ▸). In practical terms, it allows expressing our 2D mesh data by only a few Zernike coefficients applied to the polynomial basis (the Zernike polynomials). There are other benefits when using Zernike polynomials: they have some physical meaning, as most of them are associated with a usual aberration (*e.g.* spherical aberration, coma, *etc*); and they are orthonormal, thus facilitating the expansion of any profile by just projecting onto the bases (Mahajan, 2011 ▸). In this expansion, the coefficients are non-correlated. Zernike coefficients are often used in deep learning experiments in optics to parametrize the aberrations, typically for wavefront sensing [*e.g.* Saha *et al.* (2020 ▸)] or in the alignment of the optics [*e.g.* with Kirkpatrick-Baez mirrors (Luiz *et al.*, 2022 ▸)].

Error profile samples are created by defining a set of Zernike coefficients with random values. In our case, using Noll notation (Noll, 1976 ▸), we consider the first 15 polynomials excluding the four first ones (piston, horizontal tilt, vertical tilt and defocus) but adding the secondary and tertiary spherical aberrations (Noll numbers 22 and 37). In our case, for 1D simulations in the vertical plane, we are not interested in those with azimuthal dependency, thus ending with seven polynomials^2^ [6, 8, 10, 11, 14, 22, 37] – these include astigmatism, trefoil, coma, quadra­foil and primary spherical aberrations. For each one, a random coefficient should be created. Instead of applying uniform sampling for all of them in the same interval [as done by Saha *et al.* (2020 ▸)], we prefer to customize the ranges and distributions for using empirical experience. We thus sample coefficients using the distributions [n, n, n, u, n, u, u] (n = normal, u = uniform) and intervals [σ = 0.5, σ = 0.5, σ = 0.5, ±2.3, σ = 0.05, ±1.0, ±0.5] × *F* micrometres, with the factor *F* = 5. We sampled *N*_P_ 2D mesh surfaces and wrote the vertical profile to a file, to be used in our wavefront simulations.

The Zernike coefficients are orthonormal on a domain that is a disk of radius unity. If we limit the domain to another shape (*e.g.* rectangle), or, in our case, we reduce the dimensionality (1D vertical cuts), the Zernike polynomials no longer form an orthonormal set of polynomials. To solve this inconvenience, to inject into the CNN a consistent input, we orthogonalized our base of 1D cuts of Zernike polynomials using the Gram-Schmidt method, to obtain a new orthonormal 1D basis. Therefore, the coefficients to be passed to the CNN are those of the Gram-Schmidt base, and not those of the 1D Zernike cuts. This does not change the sampled error profiles used in the wavefront simulations, but changes the target values used to train and test the CNN.

### 2.3. Deep learning system

**Figure 2:**
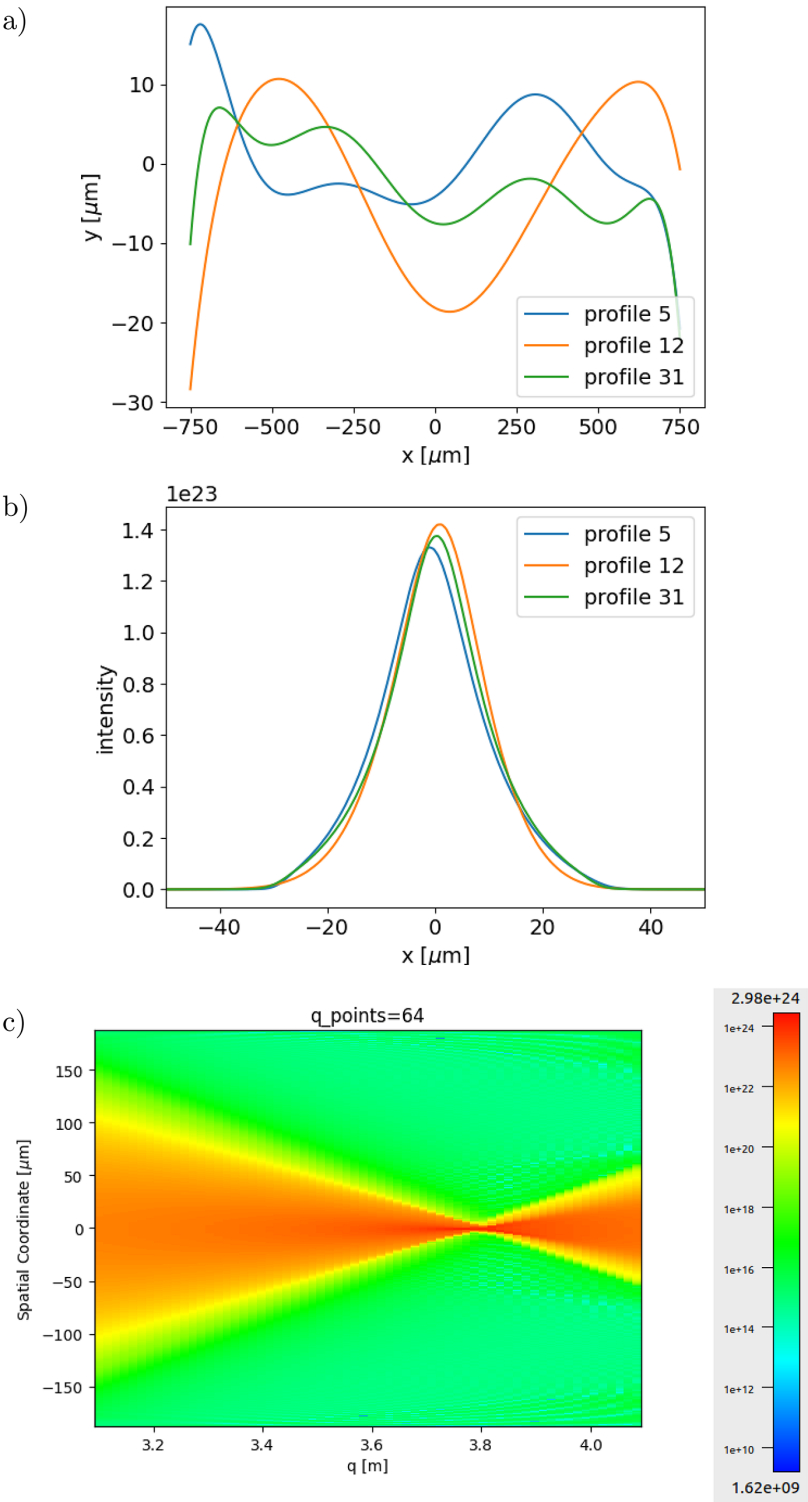
Examples of lens error profiles and intensity profiles. (*a*) Three error profiles, (*b*) their corresponding intensities at the center of the propagation interval, and (*c*) propagation or caustic plot in the full interval for error profile number 5 (color in log scale). The CRL is made of a single lens, and only the first coherence mode is used.

Once a collection of *N*_S_ sampled profiles has been prepared, the wavefront simulation is run for each one. For each sample, we calculate the intensity distribution at the *N*_P_ propagation images. Each simulated intensity plot has 1500 points. To save data volume, we reduce the number of points by interpolation to *N*_A_ = 256 points (making sure we do not miss characteristic features, structures or artifacts in the intensity distribution). Therefore, the *N*_S_ runs of the wavefront simulator produce a stack of *N*_S_ × *N*_P_ × *N*_A_ float number values, that constitute the data for the CNN. The target data is a stack of *N*_S_ × 7 values, containing the Gram-Schmidt coefficients. The data stack is saved in an .hdf5 file and the target data in a .txt file to be passed to the CNN. Running the wavefront simulations for *N*_S_ = 5000 lasted about 2 h in a CPU using a single coherent mode. For partial coherence simulations, we propagated ten coherent modes that contain more than 99% of the total intensity; therefore it takes about ten times more running time. Fig. 2 ▸ shows an example of how the data look for the first sample (defined with no deformation) and for another run. It can be appreciated that ‘big’ changes in profile always correspond to ‘small’ changes in the intensity profile. The deep learning method should be able to detect these small differences and exploit them to retrieve the correct profiles.

We constructed a CNN using *Keras* (Chollet *et al.*, 2015 ▸), inspired by the architecture of PHASENET (Saha *et al.*, 2020 ▸). Our CNN comprises five blocks stacked together. Each block contains two convolutional layers sized 3 × 3, with a stride of 1, and the number of channels doubling in each block, starting with 8. Additionally, each block includes a max-pooling layer applied only along the lateral dimensions. Following these convolutional layers, two dense layers with 64 channels each are incorporated, followed by a final dense layer with the same number of neurons as the Gram-Schmidt coefficients to be predicted (which is seven in our scenario). We utilized the ReLU activation function for all layers, except for the last layer, where linear activation was applied. This configuration results in a relatively concise CNN model containing a total of 430655 parameters.

To prevent overfitting, we looked at the accuracy of the training and validation data (a 20% fraction) and verified a uniform parallel increasing accuracy on the training and validation sets. If needed, we rerun the training with increased *N*_S_. The possibility to increase more and more *N*_S_ (*i.e.* having an unlimited number of samples) is the great advantage of using synthetic data for training the CNN, and makes the use of regularization techniques to avoid overfitting unnecessary.

We minimize the mean squared error between predicted and ground truth coefficients and train each model for *N*_E_ epochs and batch size 64 on a GPU (NVIDIA Tesla V100-SXM2-32GB) using the RMSprop optimizer with learning rate 10^−4^ for a total training time of less than 1 h.

The design and optimization of a deep learning system is more an art than a science (Chollet, 2017 ▸) and the experience is a real asset. We describe here our procedure, which follows the experience found in the literature. As discussed before, we started with a model similar to PHASENET (Saha *et al.*, 2020 ▸) with some differences to fit our needs:

(i) We use a 1D propagation model, therefore our input data has dimensions (*N*_P_, *N*_A_) = (64, 256) instead of (32, 32, 32). We then use 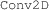 instead of 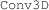 layers.

(ii) Our simulation process based on wavefront propagation is more complex and CPU-demanding; therefore it has been uncoupled from the training. Therefore, we run first the simulations in a CPU, and then the training in a GPU.

(iii) We use 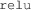 instead of 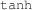 activation (a preliminary run showed much better convergence).

We use (2/3)*N*_S_ samples for training (80% for true training and 20% for validation) and (1/3)*N*_S_ for testing.

## 3. Results for the 1D propagation model

### 3.1. Results for a CRL made of a single lens

**Figure 3:**
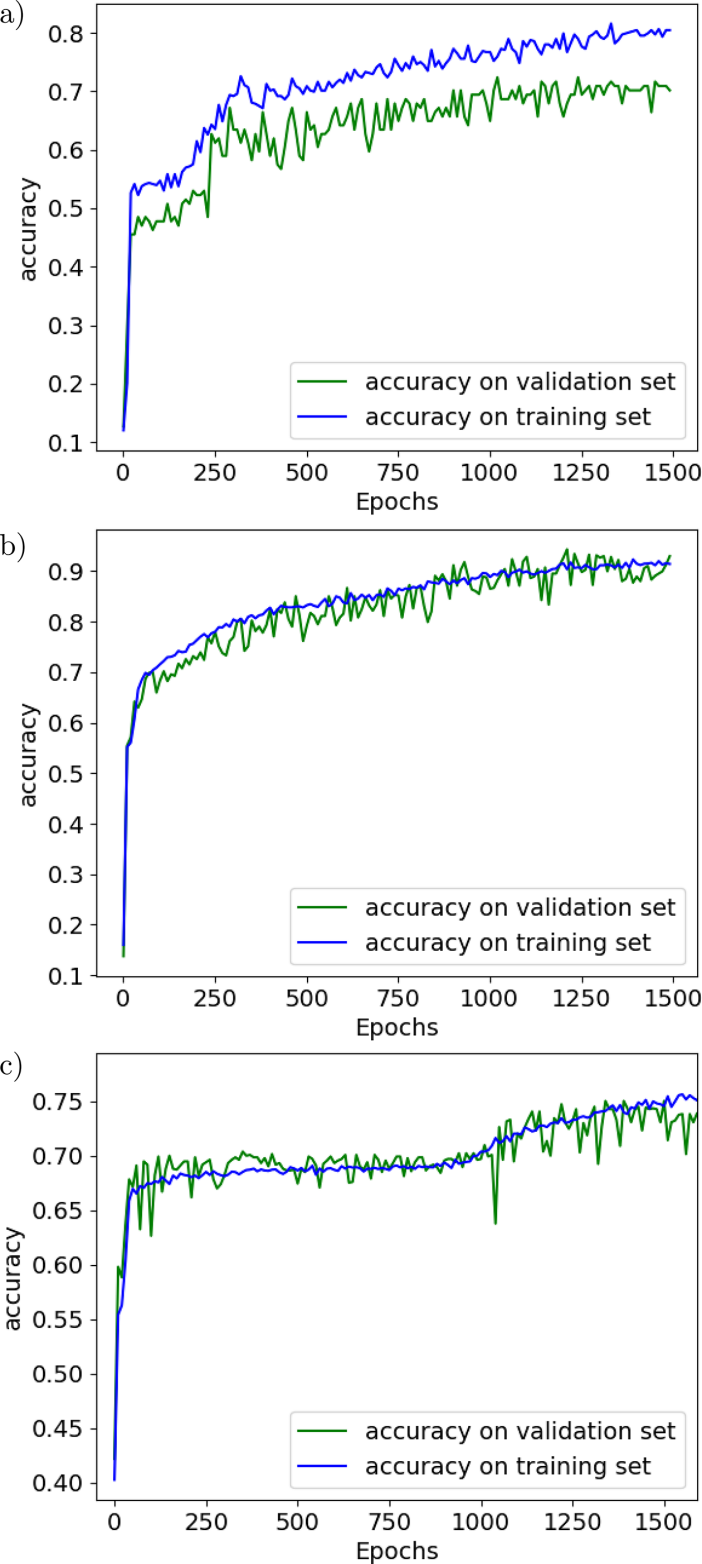
Accuracy of the training data and validation data, for (*a*) a single-lens CRL, *N*_S_ = 1000, (*b*) a single-lens CRL, *N*_S_ = 5000, and (*c*) a multi-lens CRL, *N*_S_ = 10000.

The accuracy of the training and validation data is shown in Fig. 3 ▸ versus the number of epochs. We made the first run with *N*_S_ = 1000. It can be appreciated in Fig. 3 ▸(*a*) how the learning slope reduces at about 300 epochs. The accuracy of the validation data reaches only 79%. Clearly, more samples are needed. We then run *N*_S_ = 5000 samples. The accuracy of the test data improved to 93% [Fig. 3 ▸(*b*)]. We consider that this CNN model works satisfactorily and label it as our standard configuration. Further tests will follow to study how some changes in the configuration and parameters may influence the results.

### 3.2. Results for a CRL made of ten lenses

This case implements ten lenses, with a shorter *f*. The accuracy of the training and validation data is shown in Fig. 3 ▸(*c*). A much worse accuracy (73%) as compared with the case of a single lens (93%) cn be seen. The number of samples has been raised to *N*_S_ = 10000. The reason for the worse training is due to the higher absorption of the CRL in the multi-lens CRL case: the cumulated absorption over the ten lenses reduces significantly the tails of the intensity distribution to almost zero, thus the system does not respond to changes in the error profile in this zone. This will be further discussed in the next section.

## 4. Discussion

We analyze here the influence of several parameters, concerning the learning procedure and also the influence of some physical aspects, like the use of a partially coherent beam.

### 4.1. Use of an orthonormal basis

**Figure 4:**
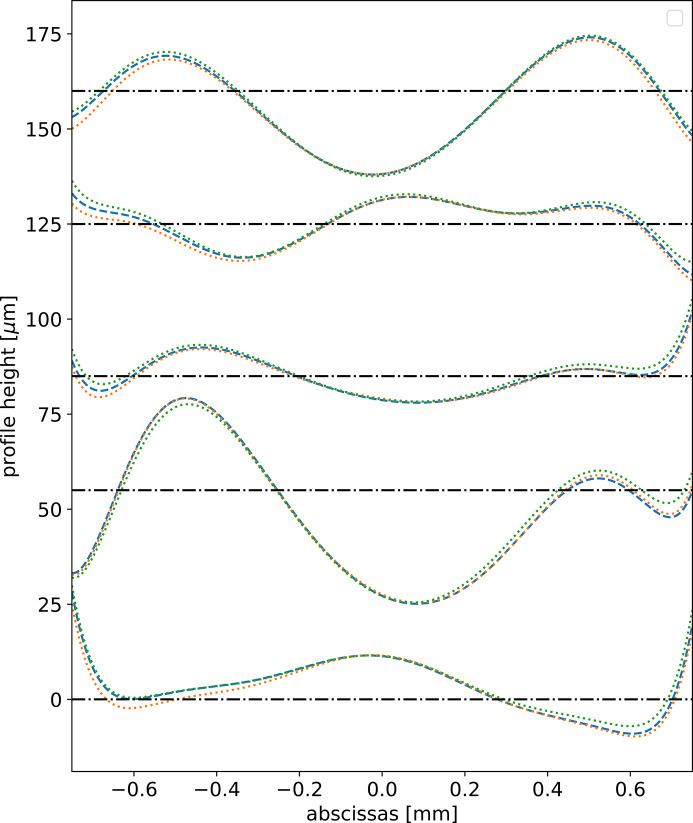
Some original and fitted (predicted) profiles from samples in the test data. In dashed blue the original profile. In dotted orange the standard model *N*_S_ = 5000 (accuracy on test data 93%) using Gram-Schmidt bases. In dotted green the same model was trained with targets using non-orthogonal 1D Zernike bases (accuracy on test data 91%). Note that, although the difference in accuracy is only 2%, there are appreciable differences in the profiles. Each profile has been shifted vertically for clarity, and the corresponding horizontal shifted axis is displayed.

The question is whether the use of an orthonormal basis for expressing the target coefficients is important. We tested the system using as targets in the training procedure the Zernike 1D coefficients instead of the Gram-Schmidt ones. As expected, the results are not so good: although accuracy is only 2% lower (91% instead of 93%), the predicted profiles agree visually less well with the true profiles (Fig. 4 ▸). However, a system using decomposition in non-orthogonal coefficients also works well.

### 4.2. Capacity of the CNN

We analyzed the possibility of reducing the capacity of the CNN. Our 1D model is much simpler than the full 2D model of Saha *et al.* (2020 ▸), thus each sample requires fewer data (we have *N*_P_ × *N*_A_ = 64 × 256 float-numbers instead of 32^3^ in PHASENET). The question is whether we can strongly reduce the capacity of the CNN. The answer is yes, but we would need more samples to obtain the same accuracy as in our standard configuration. If we remove the last convolutional block (which has the highest capacity) we obtain for the CRL system with a single lens an accuracy value of 86% (instead of 93% for the standard configuration).

### 4.3. Effect of the number of image planes and their position

Thinking about the possible experimental realization of the system discussed here, it is important to economise the number of images to be acquired (*N*_P_), and also the scanning interval. Ideally, the highest *N*_P_ and the higher the interval, the better. However, the experimental setup limits the interval, and the recording time limits *N*_P_. This is also discussed by Saha *et al.* (2020 ▸), who show that a reduction in the number of images is possible at the price of a poorer quality learning and the minimum number of images is somehow related to the number of target coefficients in use. We trained the CNN with fewer image planes by just picking the calculated data with frequency 2 (*N*_P_ = 32) and 4 (*N*_P_ = 16), resulting in an accuracy of 84% and 85%, respectively (compared with the initial 93%). We also looked at what happens if we scan the image plane out of focus. We used the calculations on the 32 planes downstream from the focus, and obtained good accuracy (92%) but with a much different learning curve with a step-down at about 700 epochs. Stopping the learning at this point, we obtained an accuracy of 90%, also showing that the system still works well.

### 4.4. Partial coherence

The quality of the intensity images (the features in our CNN) is extremely important. The presence and detectability of some structures are fundamental to retrieving the target profile. Consequently, a beam with less quality will produce worse images and therefore slow down the CNN learning (thus requiring more cycles or more data). In the limit, if the quality of the beam is too bad, the system simply does not work.

**Figure 5:**
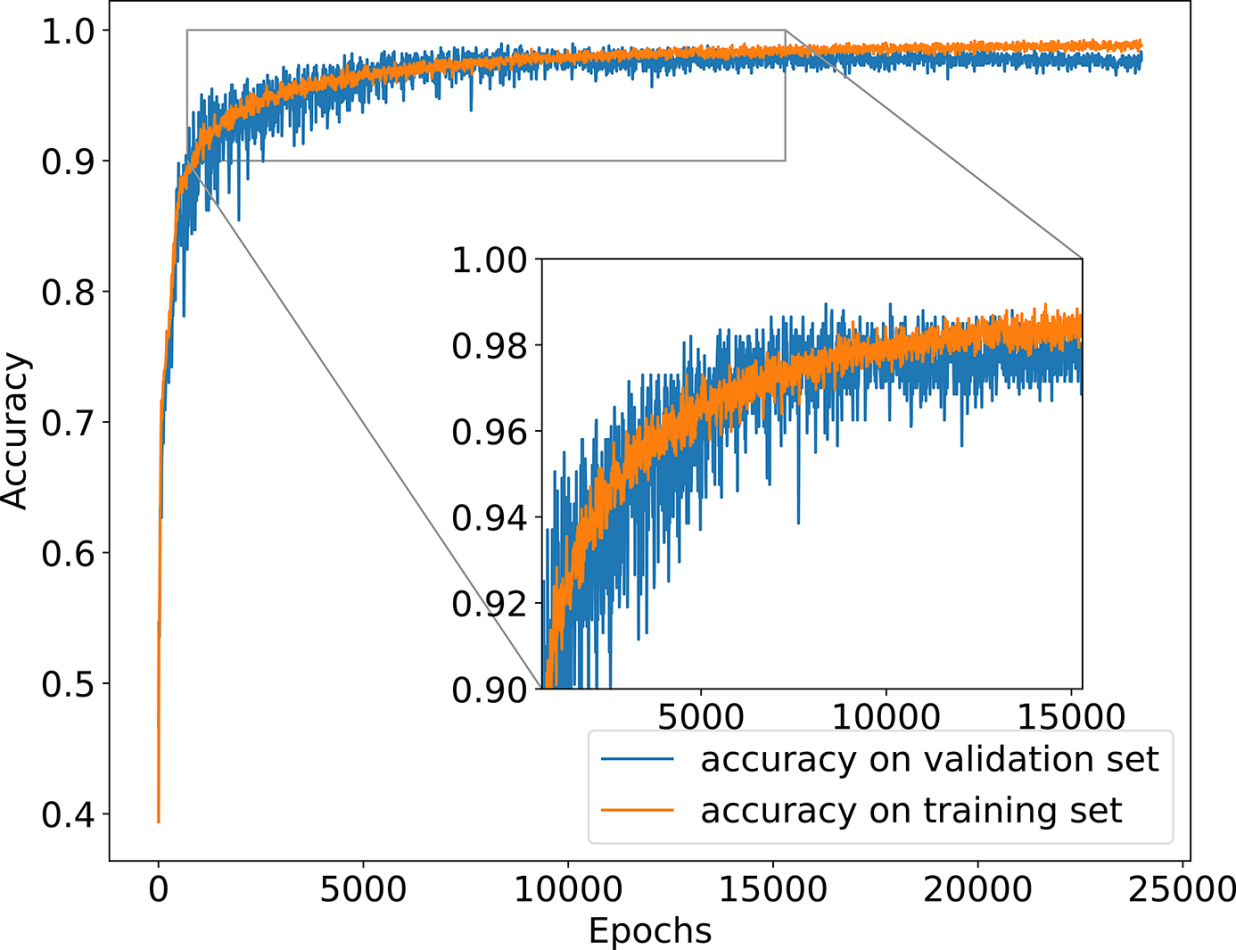
Accuracy of the training data and validation data, for partial coherence calculations using *N*_S_ = 5000, 64 planes downstream from focus, 25000 epochs. The inset shows the crossing point around 10000 epochs.

**Figure 6:**
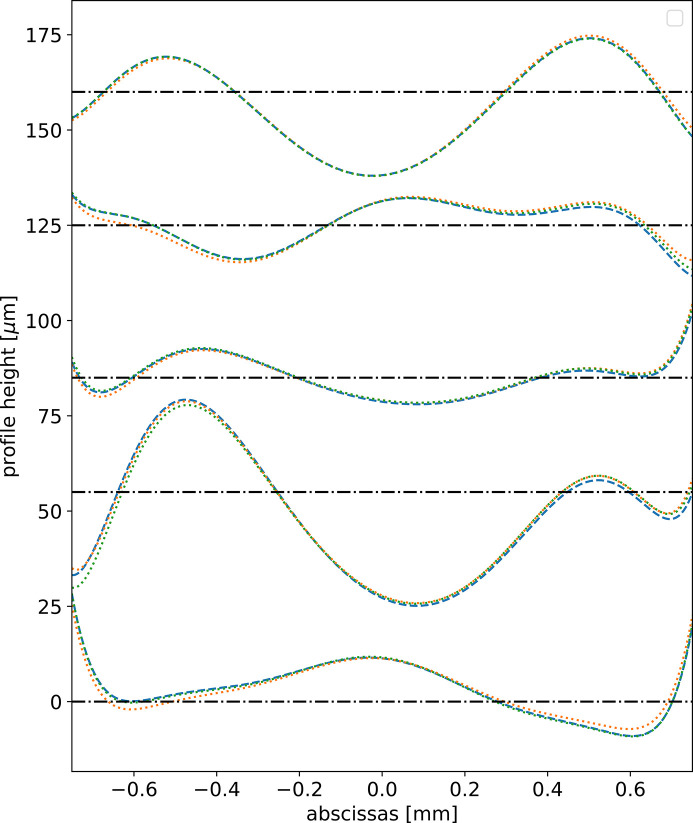
Some original and fitted (predicted) profiles from samples in the test data. We used here multimode partial coherence. In blue, the original profile; in orange, the predicted profiles using 1500 epochs (accuracy on test data 92.3%); in green, the predicted profiles using 24000 epochs (accuracy on test data 96.7%). Each profile has been shifted vertically for clarity, and the corresponding horizontal shifted axis is displayed.

Several physical factors define the quality of the beam. We are affected by the emittance and coherence. In most works related to wavefront sensors the beam is ‘prepared’ to record the point spread function. Usually, this is achieved using a pinhole that will produce something that approximates a point source. In our case, we do need a pinhole or slit, and we can use the direct synchrotron beam due to the low emittance of the fourth generation of synchrotron storage rings. The other parameter to look at is the coherence. Synchrotron radiation is not fully coherent, and is less and less coherent with increasing photon energy. In the case analyzed here, the coherent fraction in the vertical direction of the undulator source at 7 keV is about 0.6 [see Sanchez del Rio *et al.* (2022 ▸) for a full discussion], therefore the beam cannot be considered fully coherent. An analysis of the coherence can be made using coherent mode decomposition (ibid). This means that the source is decomposed into a number of coherent modes (wavefronts) that should be propagated one by one and their individual contributions added to the intensity of the image. We re-run the simulation using partial coherence. Ten modes are enough to model the partially coherent beam with high quality (representing more than 99% of its intensity). Although the volume of data created for the CNN training is the same, the calculation requires more than ten times more time. The new results are used to train the CNN with the same parameters as the standard model. In Fig. 5 ▸ we can see the learning curves, manifesting a clear underfitting, but arriving at an accuracy value of 92%. Increasing more and more (up to 25000) the number of epochs we see that the system improves. It is about *N*_E_ = 10000 when the accuracy of the training and validation sets cross. However, although the accuracy on the validation set no longer increases, it does not decrease, ending in a value of 97%. Fig. 6 ▸ shows some of these profiles for comparison.

Therefore, the use of a partially coherent beam (instead of a fully coherent beam) just slows down the learning process but it is not a limiting factor for retrieving the target coefficients with high accuracy. Although it is dangerous to extrapolate this conclusion to different systems, it is very useful to know that simulations with full coherence are good approximations to model the system. Thus, they can be used for creating synthetic training data with a much reduced computational cost.

### 4.5. Effect of the abscissas interval in the error profiles

**Figure 7:**
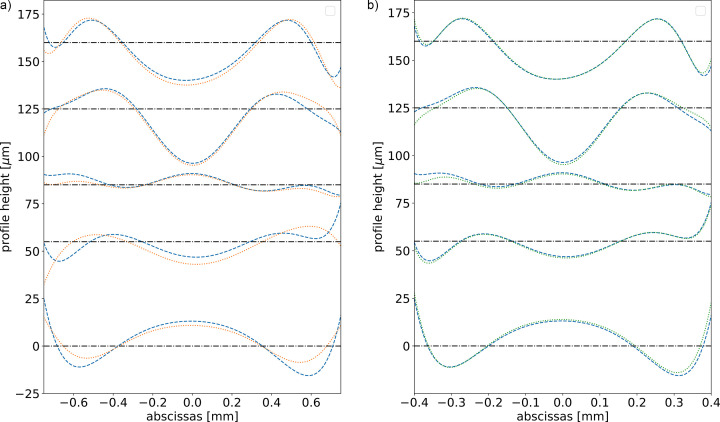
Five original (dashed blue) and fitted or predicted (dotted) profiles from samples in the test data for the multi-lens CRL case. (*a*) Error profiles defined over a window of 1500 µm; accuracy on test data 72.6%. (*b*) Error profiles defined over a window of 800 µm; accuracy on test data 84.7%. Each profile has been shifted vertically for clarity, and the corresponding horizontal shifted axis is displayed.

The learning process for the CRL with ten lenses is worse [see Fig. 3 ▸(*c*)] than for the single lens [Fig. 3 ▸(*b*)]. This is related to the illuminated area. Indeed, the larger absorption of the ten lenses means that the illumination just after the CRL is smaller for the multi-lens CRL as compared with the single-lens CRL. Obviously, the CNN is not sensitive to the changes in the error profiles in the zone that has no intensity. To test this, we adjusted the abscissas interval of the generated random profiles to better match the illuminated area. As expected, we obtained better results (see Fig. 7 ▸).

### 4.6. Zernike coefficients from full random recipe

The algorithm samples the coefficients for the different Zernike polynomials using a phenomenological model resulting from the analyses of experimental error profiles made in previous works. We tested this model against the full random model (all coefficients are created using a random uniform distribution in [0, 5 µm]). The algorithm based on the phenomenological recipe works better (72.6% accuracy) than a fully random generation of the coefficients from uniform distributions (56.2% accuracy). This is because our model weights in some way the error profile with the transmitted intensity profile. This is true for the 1500 µm window which, as discussed before, includes non-illuminated areas. If we reduce the window, both algorithms produce similar results.

### 4.7. Effect of inaccuracies in error profiles on the propagated images

**Figure 8:**
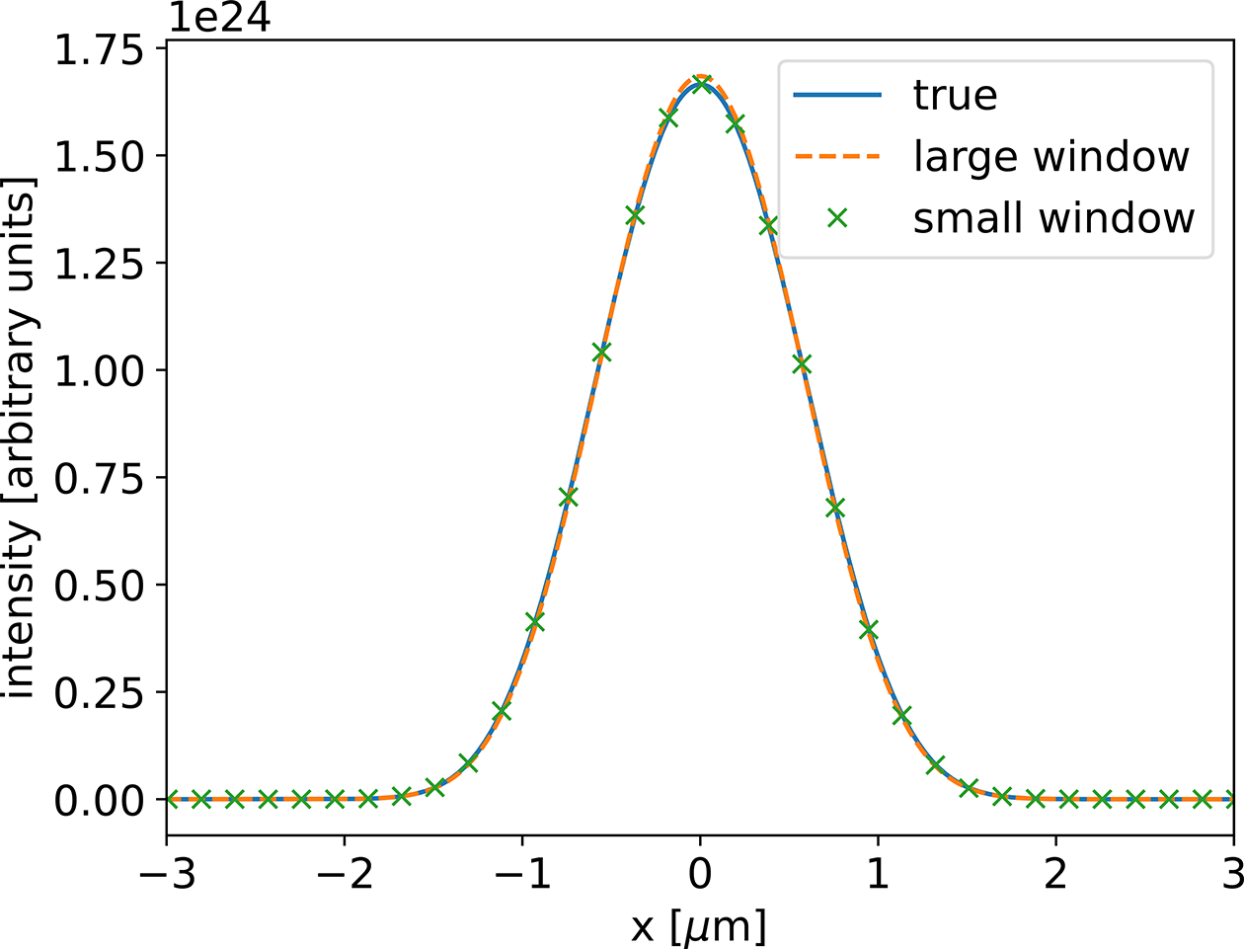
Intensity distributions for the multi-lens CRL at the central plane of the propagation interval for the middle profile in Fig. 7 ▸. The three distributions refer to the true sampled profile, the guessed profile for a window of 1500 µm, and the guessed profile for a window of 800 µm. We only observe minimum differences in the intensity distributions.

We have always measured the accuracy of the CNN by comparing the estimated error profile with the true error profile. Even when the accuracy is not excellent, for example in Fig. 7 ▸(*a*), the guessed profile usually separates from the true profile only at the edges. In these areas the transmitted intensity is low and therefore the effect of this discrepancy is small in the propagated intensity profiles. To illustrate this phenomenon, we compared the propagated wavefronts using the true error profile and the estimated error profile for the middle profile in Fig. 7 ▸. The results show a very similar intensity distribution (Fig. 8 ▸).

## 5. Conclusions

Relying solely on the intensity of the propagated beam at different distances, we have illustrated how neural networks could accurately predict the surface error of a lens system. While this methodology has been demonstrated in other fields, such as 3D microscopy [see, for example, Saha *et al.* (2020 ▸)], we not only expanded this approach into the X-ray range but also investigated the influence of the synchrotron radiation partial coherence. Furthermore, we examined the significance of utilizing aberration coefficients from an orthonormal basis to consistently train the neural networks.

The trained CNN is a robust model that works satisfactorily in many conditions. Many tuning parameters that can be changed in the CNN and also physical phenomena like the number of planes used or the effect of partial coherence have been analyzed and, although showing more or less sensitivity to the accuracy of the results, they always produce reasonably good results. This feasibility study opens the way to other more complete analyses. The next effort will consist of dealing with 2D wavefronts and images. The usefulness of the CNN trained with synthetic data and being fed with experimental images will be addressed in a future work. Last, but not least, this methodology is not restricted to refractors (our X-ray lenses) but can also be applied to any focusing system with reflectors and diffractors, and is independent of the multiple origins of the surface shape errors (fabrication process, clamping and gravity sag, thermal load deformations).

## 6. Data availability

Data underlying the results presented in this paper are publicly available at https://github.com/oasys-esrf-kit/Paper_JSR_zt5005.

## Footnotes

1 We used the electron beam sizes and divergences  = 29.7 µm,  = 4.37 µrad,  = 5.29 µm,  = 1.89 µrad, corresponding to beam emittances ɛ_*x*_ = 130 pm rad, ɛ_*y*_ = 10 pm rad and beta functions β_*x*_ = 6.8 m, β_*y*_ = 2.8 m.

2 In fact, polynomial 12 has been removed as it looks to be easily represented by the others (in the Gram-Schmidt process this was reproduced with a lot of noise).
